# Is Merkel cell carcinoma associated with high and chronic arsenic dose exposure?^⋆^

**DOI:** 10.1016/j.abd.2022.06.013

**Published:** 2023-12-21

**Authors:** Irving Llibran Reyna-Rodríguez, Valeria F. Garza-Davila, Jorge Ocampo-Candiani, Sonia Chavez-Alvarez

**Affiliations:** Dermatology Department, Universidad Autonoma de Nuevo Leon, Facultad de Medicina y Hospital Universitario “Dr. José E. Gonzalez”, Monterrey, Mexico

Dear Editor,

Merkel cell carcinoma (MCC) is a highly aggressive primary cutaneous tumor of neuroendocrine origin. It occurs predominantly in Caucasian male adults in photo-exposed areas. Despite being a rare tumor with an incidence of 0.1–1.6 cases per 100,000 habitants, diagnostic yield has increased these numbers.^1^ We present a Hispanic man in his 70 s from the Northern region of Mexico, known for high levels of arsenic in its water, presented at our clinic for evaluation of a localized dermatosis. He had a family history of maternal breast cancer and stomach cancer in his 2 sisters. Physical examination revealed multiple painless and rapidly growing flesh-colored and red-violaceous nodules on the right axillary region (Fig. 1). This began 2 months prior, accompanied by weight loss and fatigue. He had not sought medical attention before. Dermoscopic findings (polarized light) demonstrated milky pink and white structureless areas. An excisional skin biopsy was performed (Fig. 1). Histopathology revealed a flattened epidermis due to a nodular infiltrate located in the papillary and reticular dermis. At a higher magnification, the cluster of cells appeared monotonous with an epithelioid/lymphomyeloid appearance with abundant mitosis. Most cells had a loss of the nucleus-to-cytoplasm ratio, but they still retained an abundant cytoplasm with a prominent nucleus. Immunohistochemistry stained positive for AE1/AE3, CK20 (Fig. 2), and synaptophysin. Negative immunohistochemistry included CK7, SOX-10, S100, HMB45, CD45, TTF-1, vimentin. The clinical, pathological and immunohistochemical findings were consistent with Merkel cell carcinoma. Merkel cell carcinoma has been associated with exposure to ultraviolet radiation, immunosuppression, and polyomavirus infection.^2^ Diagnosis is made with immunohistochemistry which also helps make a distinction from histologically similar tumors. Dermatopathology shows a nodular or diffuse infiltrate composed of small blue cells with hyperchromatic nuclei and scarce cytoplasm. Mitoses are frequently abundant, and apoptosis is often widespread. 2 AE1/AE3, CK20, synaptophysin chromogranin, neuron-specific enolase and neurofilament stains are positive; CK7, TTF1, CDX2, S100, CD45, and vimentin are negative.^2^ The main differential diagnoses before immunohistochemistry include metastatic neuroendocrine carcinoma (TTF1+, CK7+, CK20−), small cell melanoma (S100+, Melan-A/MART1+, HMB45*, SOX10*, vimentin+, CK20−) and lymphoma (CD45+, CD43+, CD3+, CD20+, CK20−, chromogranin−, synaptophysin−).^2^ Exposure to high rates of arsenic in the environment or from contaminated water has been associated with an increased incidence of malignancies. Very few MCC cases (a total of 14) have been associated with arsenic.^3^, ^4^, ^5^ The treatment of choice in the early stages is surgical excision accompanied by radiotherapy. In advanced stages, there is no established curative therapy, relying on palliative chemotherapy.^2^ It has a low survival rate even when tumors are localized or treated with new therapies such as immunotherapy targeting Programmed cell Death-1 (PD-1) or its Ligand (PD-L1).^1^ Our patient refused to receive any therapies. He developed cutaneous metastasis and involvement of internal organs in one month and died two months later. The patient’s family and personal history of malignancies made us rethink the relationship between the environment in his native region and the development of Merkel carcinoma. They were native to Torreon, Coahuila, Mexico, a geographic zone with high arsenic levels. More objective evidence is required regarding this possible association. We intend to awaken an interest on Merkel pathogenesis and environmental factors.

**Figure 1:**
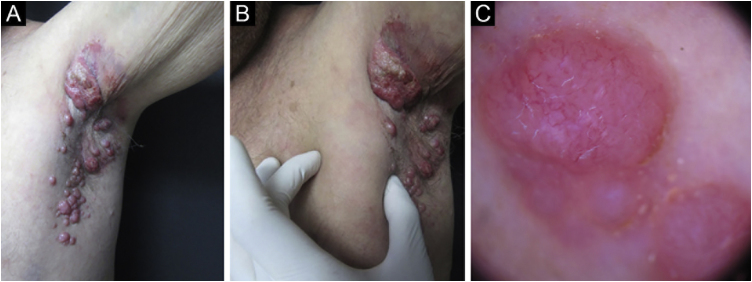
(A) Clinical image showing multiple flesh-colored nodules in the right axillary region. (B) The lesion a couple of weeks after initial presentation. (C) Dermoscopy of the axillary nodules showing irregular vessels.

**Figure 2:**
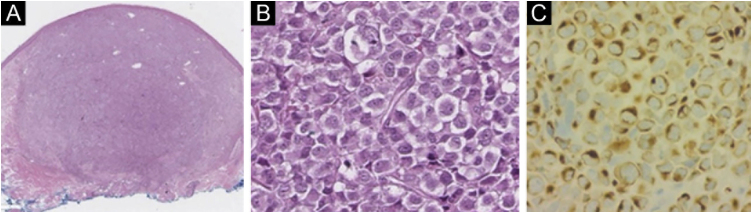
(A) Specimen (10× magnification) with hematoxylin-eosin showing a well-defined non-encapsulated tumor in the dermis (Hematoxylin eosin). (B) A magnification (200×) with hematoxylin-eosin showing small to medium monotonous cells with an epithelioid/lymphomyeloid appearance and abundant mitosis. (C) CK20 (200× magnification) immunohistochemistry in perinuclear dot-like pattern.

## Financial support

None declared.

## Authors’ contributions

Irving Llibran Reyna-Rodríguez: The study concept and design; data collection, or analysis and interpretation of data; writing of the manuscript or critical review of important intellectual content; data collection, analysis and interpretation; intellectual participation in the propaedeutic and/or therapeutic conduct of the studied cases; final approval of the final version of the manuscript.

Valeria F Garza-Davila: Data collection, or analysis and interpretation of data; data collection, analysis and interpretation; effective participation in the research guidance; intellectual participation in the propaedeutic and/or therapeutic conduct of the studied cases; final approval of the final version of the manuscript.

Jorge Ocampo-Candiani: Data collection, analysis and interpretation; effective participation in the research guidance; critical review of the literature; final approval of the final version of the manuscript.

Sonia Chavez-Alvarez: The study concept and design; data collection, or analysis and interpretation of data; writing of the manuscript or critical review of important intellectual content; data collection, analysis and interpretation; effective participation in the research guidance; intellectual participation in the propaedeutic and/or therapeutic conduct of the studied cases; critical review of the literature; final approval of the final version of the manuscript.

## Conflicts of interest

None declared.

## References

[1] Freeman M.B., Holman D.M., Qin J., Lunsford N.B. (2019). Merkel cell carcinoma incidence, trends, and survival rates among adults aged ≥50 years from United States cancer statistics. J Am Acad Dermatol..

[2] Coggshall K., Tello T.L., North J.P., Yu S.S. (2018). Merkel cell carcinoma: an update and review: pathogenesis, diagnosis, and staging. J Am Acad Dermatol..

[3] Ho S.Y., Tsai Y.C., Lee M.C., Guo H.R. (2005). Merkel cell carcinoma in patients with long-term ingestion of arsenic. J Occup Health..

[4] Chou T.C., Tsai K.B., Wu C.Y., Hong C.H., Lee C.H. (2016). Presence of the Merkel cell polyomavirus in Merkel cell carcinoma combined with squamous cell carcinoma in a patient with chronic arsenism. Clin Exp Dermatol.

[5] Choudhury M.I.M., Shabnam N., Ahsan T., Ahsan S.M.A., Kabir M.S., Khan R.M. (2018). Cutaneous malignancy due to arsenicosis in Bangladesh: 12-Year study in tertiary level hospital. Biomed Res Int..

